# Effect of Practicing Health Behaviors on Unmet Needs among Patients with Chronic Diseases: A Longitudinal Study

**DOI:** 10.3390/ijerph18157977

**Published:** 2021-07-28

**Authors:** Bich-Na Jang, Hwi-Jun Kim, Bo-Ram Kim, Seonyeong Woo, Woo-Jin Lee, Eun-Cheol Park

**Affiliations:** 1 Department of Public Health, Graduate School, Yonsei University, Seoul 03722, Korea; jbn2846@gmail.com (B.-N.J.); woojzn11@yuhs.ac (W.-J.L.); 2 Institute of Health Services Research, Yonsei University, Seoul 03722, Korea; 3 Operation Branch, 27th Infantry Division Medical Battalion, Chuncheon-si 24251, Korea; kimhwijun88@yuhs.ac; 4 Health Promotion Branch, 11th Infantry Division Medical Battalion, Hongcheon-gun 25141, Korea; amirrami@naver.com; 5 Faculty of Nursing, Korea Armed Forces Nursing Academy, Dajeon 34059, Korea; wsy2730@naver.com; 6 Department of Preventive Medicine, Yonsei University College of Medicine, Seoul 03722, Korea

**Keywords:** health behavior, chronic disease, unmet need

## Abstract

With the growing prevalence of chronic diseases, the proportion of unmet needs is increasing. In this study, we investigated the effect of practicing health behaviors on unmet needs among patients with chronic diseases, using data from the Korea Health Panel Survey conducted between 2014–2017. Participants (*n* = 4069) aged 19 or older, with at least one chronic disease (hypertension, diabetes mellitus, dyslipidemia, or arthrosis) and with existing follow up data were selected. Health behaviors combined three variables: not presently smoking, not belonging to high-risk drinking group, and indulging in moderate- or high-intensity exercise. Those who met all three criteria were classified as the practicing health behaviors group. Generalized Estimating Equation analysis was performed to consider correlated data within a subject. Of the participants, 23.9% practiced health behaviors. Participants who did not practice health behaviors were significantly more likely to have unmet needs compared with those who did (OR: 1.24, 95% CI: 1.10–1.39). Further research would be needed to verify the impact of practicing health behavior on unmet needs.

## 1. Introduction

### 1.1. Introduction

The global phenomenon of chronic diseases or non-communicable diseases is increasing in proportion due to genetic, physiological, environmental, and behavioral factors [1,2,3]. In South Korea, the prevalence of chronic diseases is increasing because of the growing proportion of the elderly population, poor health behaviors, etc. [2]. The World Health Organization (WHO) suggests that behaviors such as smoking, drinking, consuming excessive sodium, and insufficient physical activity are risk factors for chronic diseases [1]. However, chronic diseases can be prevented or managed by adopting healthy behaviors [1].

Therefore, many governments are conducting a campaign to improve lifestyle at the national level and implementing a primary care-oriented management project for patients with chronic diseases. Several countries enhance the role of general practitioners in promoting community health networks [4,5]. However, in South Korea, since the community-centered primary care system is insufficient, delivery system in healthcare services is mainly provided by clinics and hospitals [6]. Therefore, visiting clinics and hospitals would have a significant effect on meeting the medical needs of patients with chronic diseases.

Patients with chronic diseases experience an increase in individual medical needs as the severity increases [7], but the medical services required to support them may not be available. Unmet health care needs occur when people need medical services but are unable to receive appropriate medical care resulting from factors such as financial deficiency and lack of availability of services [8]. Unmet needs considers both medical needs and medical satisfaction of individuals [9].

### 1.2. Background

According to a previous study, the rate of unmet needs is decreasing every year in South Korea, but as the age increases, the unmet needs rate is increasing [10]. Thus, it is difficult to mitigate the rate of unmet needs because of the increasing elderly population [11] and prevalence of multimorbidity, which increases proportionally with age [12]. A study identifying the degree of unmet needs for the elderly showed that their rate of unmet needs was higher than that of the entire population, which indicates barriers in the Korean health care system [13].

Several studies have reported that people with chronic diseases are more likely to experience unmet needs [14,15,16]. One study comparing the rate of unmet needs between 1998 and 2017 revealed that participants who had chronic diseases experienced more unmet needs in 2017 than they did in 1998, although insurance coverage and access to services had been improved [16]. In addition, according to another study, the more the adults had chronic conditions, the greater they experienced unmet needs [14]. Therefore, it can be inferred that relieving patients’ symptoms of chronic diseases might mitigate their unmet needs.

Meanwhile, a study reported that chronic disease patients with higher income had greater acceptance of recommendations for health behavior change than chronic disease patients with lower income, due to barriers such as cost and access [17]. It is also suggested that enough financial support for health promotion may lead to improved health conditions and reduced unmet needs. Another study examining pre-post effectiveness of evaluation of chronic disease management yielded positive results for patients with fewer visits to the clinic [18]. However, there are insufficient studies that examine the relationship of an improved lifestyle to unmet needs among patients with chronic diseases.

### 1.3. Purpose

This study bridges this research gap by examining the effect of practicing health behaviors on unmet needs. We hypothesized that there will be a difference in the level of unmet needs when patients with chronic diseases practice healthy behaviors. In addition, we aim to investigate the effect of different health behaviors on unmet needs and identify their relationship after categorizing for the type of chronic disease.

## 2. Materials and Methods

### 2.1. Study Population

**Figure 1:**
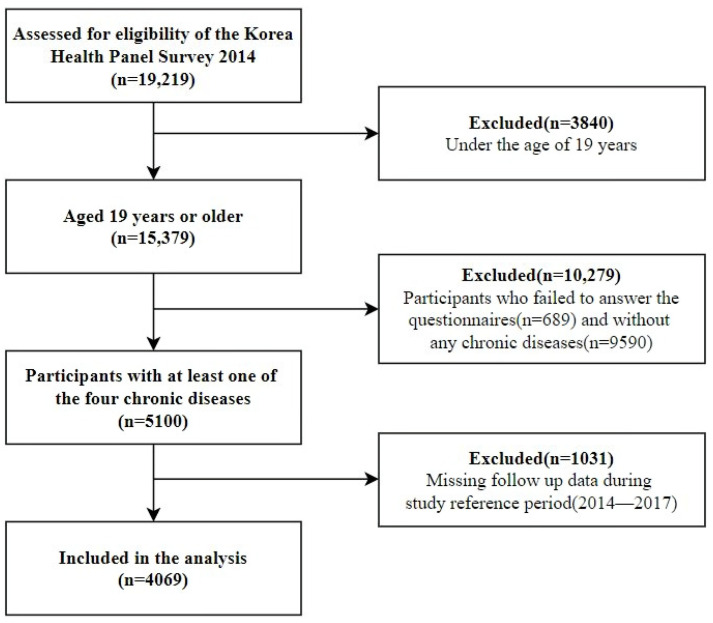
Flowchart of the participant selection.

This study used data from a secondary data source, the Korea Health Panel Survey (KHPS) conducted between 2014–2017. This data is gathered for households annually by the Korea Institute for Health and Social Affairs and the National Health Insurance Service since 2008. The database does not contain any personal information on participants. This study was reviewed and approved by the Institutional Review Board of Yonsei University’s Health System (IRB number: 4-2021-0121). Since chronic diseases have different definitions and classifications, we selected the top four disease diagnoses (hypertension, diabetes mellitus, dyslipidemia, arthrosis) with the highest frequencies, when classified according to the Korean Classification Disease codes (KCD codes), for our study reference period (2014–2017). In 2014, there were 5100 participants aged 19 or older with at least one of the four chronic diseases. After excluding those with missing follow up data, the final baseline population included in our study was 4069 participants. A flow chart of the participant selection is shown in Figure 1.

### 2.2. Variables 

The outcome variable was unmet needs. This was measured using the following question: “In the past year, have you ever had a need to visit a clinic or a hospital for treatment or examination, but not received appropriate care (except dental services)?” Participants responded with “yes” if they had experienced this more than once, and with “no” if they did not have to receive any treatment or examination.

The primary independent variable in this study was practicing health behaviors. To define health behavior, three variables were combined: not presently smoking, not belonging to a high-risk drinking group, and indulging in moderate- or high-intensity exercise. Those who met all three criteria were categorized in the practicing health behaviors group. Otherwise, participants were categorized in the non-practicing health behaviors group.

When categorizing smoking or not, we used pack-year to consider the quantity of smoked cigarettes. Those who smoked more than one pack-year were classified as smokers based on previous studies. Practicing moderate- or high-intensity exercise was classified according to the physical activity guidelines for South Koreans. The guideline varies between adolescents, adults, and the elderly.

Other independent variables included sex, age, marital status, region, educational level, economic activity status, household income, medical coverage type, private health insurance, Charlson Comorbidity Index (CCI), number of chronic diseases, body mass index (BMI), and medication adherence. The number of chronic diseases refers to the number of diagnosed diseases among the four chronic diseases included in this study. BMI is classified into three categories according to guidelines of the Korean Society for the Study of Obesity. Medication adherence is the extent of the patient’s drug compliance as prescribed for the four chronic diseases. If participants did not take any medication for chronic diseases, they were classified as “not applicable”. In addition, CCI was included to adjust the severity of the comorbid diseases. CCI is the sum of weighted scores for 16 groups of diseases. The higher the score, the higher the severity.

### 2.3. Statistical Analysis

To identify the general characteristics of the study subjects at the base-line year (2014), we performed a Chi-squared test. In addition, we analyzed the effects of the independent variables on dependent variables including the main independent variables through one of the panel analyses, Generalized Estimating Equation (GEE). This approach is a method for extending the generalized linear model using quasi-likelihood estimation, which identifies the effect of practicing health behaviors and other covariates on dependent variables [19]. We also examined the effect of type of health behavior on unmet needs and also performed analysis regarding the effect of practicing health behaviors on unmet need categorized by types of chronic diseases. For all values calculated in our study, a *p* value of <0.05 was considered statistically significant. All statistical analyses were performed using SAS software (version 9.4, SAS Institute, Cary, NC, USA).

## 3. Results

Table 1 shows the general characteristics and distribution of the study population. Of a total of 4096 participants included in 2014, the mean ± standard deviation of the participants’ age was 65.9 ± 10.7 years (distribution range: 29–96 years). Among them, 972 individuals (23.9%) were categorized in the group practicing health behaviors. Among those who responded as experiencing unmet needs (634, 15.6%), 142 individuals (14.6%) were in the group practicing health behaviors, and 492 individuals (15.9%) were included in the group not practicing health behaviors. However, this was not statistically significant (*p*-value = 0.3643).

Table 2 presents the results of GEE analyzing the effect of practicing health behaviors on unmet needs. Compared to those practicing health behaviors, participants who did not practice health behaviors had significantly higher association with unmet needs (OR: 1.24, 95% CI: 1.10–1.39).

Table 3 presents the results of GEE analyzing the effect of type of practicing health behavior on unmet needs. In general, the higher the number of unpracticed health behaviors, the higher association with unmet needs compared to practicing all three health behaviors. Especially, participants who only did not exercise (OR: 1.22, 95% CI: 1.08–1.38), participants who only smoked and did not exercise (OR: 1.47, 95% CI: 1.19–1.81), participants who only smoked and drank alcohol (OR: 1.70, 95% CI: 1.23–2.36), and participants who did not practice any of the three health behaviors (OR: 1.68, 95% CI: 1.24–2.27) were significantly associated with experiencing unmet needs.

Table 4 shows the results of GEE analyzing the effect of practicing health behaviors on unmet needs categorized based on the type of chronic disease. We classified type of chronic disease into seven categories in order of the highest frequency. During the study period, less than 1000 participants were classified as belonging to the “Others” type of chronic disease. The number of participants with each type of chronic disease are also shown in Table 4. The most frequent disease type was hypertension, and was significantly associated with unmet needs when not-practicing health behaviors (OR: 1.36, 95% CI: 1.04–1.79). In addition, participants who had hypertension and diabetes and did not practice health behaviors were significantly associated with experiencing unmet needs (OR: 2.40, 95% CI: 1.37–4.23). Those who were classified as “Others” and did not practice health behaviors were more significantly associated with unmet needs (OR: 1.42, 95% CI: 1.26–1.75).

## 4. Discussion

This study aimed to investigate the effect of practicing health behaviors on unmet needs among patients with at least one of the four most frequently occurring chronic diseases. GEE analyses showed that the participants who did not practice health behaviors experienced unmet needs 1.24 times more than those practicing health behaviors. This result had statistical significance. This means that those who practiced health behaviors received fewer medical services than needed. Previous studies have shown that the worse the subjective health condition, the higher the unmet needs [20,21]. One study proved that unmet needs were related to participants’ poor condition and health-related quality of life (HRQoL) [22]. Moreover, a positive association was indicated between poor HRQoL, unhealthy life style, and unmet needs among individuals with multimorbidity in another study [23]. One study reported negative outcomes of unmet needs, which is likely to lead to the higher number of multiple chronic conditions among the elderly [24]. This suggests that poor healthy lifestyles are related to unmet needs, and this is associated with a decline in self-related health condition. Since these factors are closely related to each other, it is important to practice health behaviors.

In addition, a study showed that those who practiced health behaviors experienced improved self-efficacy and decreased health distress than those who did not [25]. This leads to better subjective health status and decreases the likelihood of experiencing unmet needs. Moreover, the effect of several particular types of health behavior on unmet needs does not yield a complete linear relationship. However, those who did not practice health behaviors experienced a significant increase in unmet needs, compared with those who did practice all three health behaviors. This result can also be identified in a similar context to the previous one.

Regarding the effect of practicing health behaviors on unmet needs based on the type of chronic disease, patients with hypertension only and patients with both hypertension and diabetes were more likely to experience unmet needs if they did not practice health behaviors. We would like to look at the reasons for this in connection with the chronic disease management project. Hypertension and diabetes are the most common chronic diseases in South Korea. The primary chronic disease management pilot project, which was based on the Chronic Care Model (CCM), to provide comprehensive community-oriented prevention services for these two diseases, has been carried out since 2014 [26]. Although this project has achieved results such as increased continuity of care, increased primary healthcare utilization, and improved clinical outcomes for participants, the limitations include low rate of education completion, low enrollment rate of patients under 65 years of age, and insufficient role establishment of participating medical institutions [27]. Studies have shown that CCM has improved health conditions and reduced healthcare utilization of participants [28,29,30], but problems such as lack of connections between the central government and the community, lack of integrated management, and ambiguity of professionals’ roles resulted in unmet needs for patient with hypertension or diabetes [27]. In addition, there is a possibility that patients with chronic diseases experience unmet needs because continuous management, such as correction of unhealthy habits, has not been carried out [31].

This study has several strengths. First, it did not need to make any assumptions about missing variables, since we excluded participants who had missing data during the study period (2014–2017). Thus, the findings accurately explain the effect of practicing health behaviors on unmet needs and healthcare cost and utilization. Second, the results are universally applicable because the chronic diseases included in this study are four of the most frequently occurring. Moreover, the CCI score and the number of chronic diseases were included as covariates to compensate for the severity of individual diseases. Finally, since the health behaviors included in this study were analyzed by classifying whether all three health behaviors (smoking, drinking, and exercising) were practiced simultaneously, we can detect how practicing multiple health behaviors at the same time affects unmet needs.

Despite the advantages, this study has some limitations. First, since it is an observational study, it is difficult to control the subjects and is less reliable than RCT studies, which compare results between intervention and control groups. Similarly, the findings may have been under-represented as there may have been other factors involved (e.g., poor health, secondhand smoking, psychological issues, and other environmental changes) for subjects who had not practiced health behaviors and then started to practice them. Second, this study did not include dietary conditions, one of the main health behaviors, due to data limitations. Thus, we added BMI as a covariate, as this indicates participants’ obese status [32]. Third, for continuous follow-up observations, the data of excluded subjects may affect the results of the study, although this may result in omission bias. However, since imputation for missing values can differ from real data, the analysis results can still be accurate using only target data with all trace data [33]. Fourth, practicing health behaviors may not be a good way to promote health status for some patients. For example, diabetic patients with a diabetic foot cannot perform physical activity regularly. Finally, due to data limitation, it is unclear whether patients experienced actual improved clinical outcomes.

## 5. Conclusions

In this study, we found that patients with chronic diseases who did not practice health behaviors tended to experience higher levels of unmet needs compared with those who did. In addition, specific health behaviors and specific chronic diseases were associated with unmet needs. Further research is needed to verify the effect of practicing health behavior on unmet needs.

## Figures and Tables

**Table 1:** General characteristics of the study population categorized by unmet needs at the 2014 baseline.

Variables	Unmet Needs						
	TOTAL		Yes		No		*p*-Value
	N	%	N	%	N	%	
Total (N = 4069)	4069	100.0	634	15.6	3435	84.4	
Practicing health behaviors							0.3643
Yes	972	23.9	142	14.6	830	85.4	
No	3097	76.1	492	15.9	2605	84.1	
Age (years)							0.0548
<60	1090	26.8	155	14.2	935	85.8	
60–69	1293	31.8	189	14.6	1104	85.4	
≥70	1686	41.4	290	17.2	1396	82.8	
Sex							<0.0001
Men	1689	41.5	216	12.8	1473	87.2	
Women	2380	58.5	418	17.6	1962	82.4	
Marital Status							<0.0001
Living with spouse	2973	73.1	395	13.3	2578	86.7	
Living without spouse	1096	26.9	239	21.8	857	78.2	
Region							0.4178
Urban	1570	38.6	235	15.0	1335	85.0	
Rural	2499	61.4	399	16.0	2100	84.0	
Educational level							<0.0001
Middle school or less	2504	61.5	448	17.9	2,056	82.1	
High school	1046	25.7	126	12.0	920	88.0	
College or over	519	12.8	60	11.6	459	88.4	
Economic activity status							0.7274
Yes	2070	50.9	318	15.4	1752	84.6	
No	1999	49.1	316	15.8	1683	84.2	
Household income (quintile)							<0.0001
Lowest	1137	27.9	264	23.2	873	76.8	
Mid-low	927	22.8	149	16.1	778	83.9	
Middle	807	19.8	101	12.5	706	87.5	
Mid-high	641	15.8	65	10.1	576	89.9	
Highest	557	13.7	55	9.9	502	90.1	
Medical aid							<0.0001
Yes	220	5.4	59	26.8	161	73.2	
No	3849	94.6	575	14.9	3274	85.1	
Private health insurance							0.3064
Yes	576	14.2	81	14.1	495	85.9	
No	3,493	85.8	553	15.8	2940	84.2	
Charlson comorbidity index							0.6796
0	2715	66.7	425	15.7	2290	84.3	
1	1004	24.7	160	15.9	844	84.1	
≥2	350	8.6	49	14.0	301	86.0	
The number of chronic diseases							0.1674
1	2092	51.4	305	14.6	1787	85.4	
2	1364	33.5	231	16.9	1133	83.1	
≥3	613	15.1	98	16.0	515	84.0	
Body mass index (kg/m^2^)							0.7758
Underweight & Normal range	1510	37.1	239	15.8	1271	84.2	
Overweight	1197	29.4	179	15.0	1018	85.0	
Obese	1362	33.5	216	15.9	1146	84.1	
Medication adherence							0.0002
Good	3410	83.8	496	14.5	2914	85.5	
Poor	358	8.8	78	21.8	280	78.2	
Not applicable	301	7.4	60	19.9	241	80.1	

**Table 2:** Results of GEE analyzing the effect of practicing health behavior on unmet needs.

Variables	Unmet Needs	
	OR	95% CI
Practicing health behaviors		
Yes	1.00	
No	1.24	(1.10–1.39)
Age (years)		
<60	1.00	
60–69	0.78	(0.67–0.91)
≥70	0.72	(0.61–0.85)
Sex		
Men	1.00	
Women	1.22	(1.08–1.39)
Marital Status		
Living with spouse	1.00	
Living without spouse	1.50	(1.33–1.69)
Region		
Urban	1.00	
Rural	1.04	(0.93–1.16)
Educational level		
Middle school or less	1.25	(1.02–1.55)
High school	1.07	(0.87–1.33)
College or over	1.00	
Economic activity status		
Yes	1.00	
No	0.89	(0.79–0.99)
Household income (quintile)		
Lowest	2.14	(1.76–2.61)
Mid-low	1.50	(1.24–1.81)
Middle	1.19	(0.98–1.45)
Mid-high	1.09	(0.89–1.33)
Highest	1.00	
Medical aid		
Yes	1.42	(1.17–1.73)
No	1.00	
Private health insurance		
Yes	1.00	
No	1.03	(0.88–1.21)
Charlson comorbidity index		
0	1.00	
1	0.99	(0.88–1.11)
≥2	0.99	(0.84–1.17)
The number of chronic diseases		
1	1.00	
2	1.07	(0.95–1.20)
≥3	1.09	(0.94–1.25)
Body mass index (kg/m^2^)		
Underweight & Normal range	1.00	
Overweight	0.88	(0.79–0.99)
Obese	0.89	(0.79–1.00)
Medication adherence		
Good	1.00	
Poor	1.59	(1.34–1.89)
Not applicable	1.33	(1.10–1.59)

**Table 3:** Results of GEE analyzing the effect of type of practicing health behaviors on unmet needs *.

Variables	Unmet Needs	
	OR	95% CI
Practicing health behaviors		
no SMK & no DRK & EXC	1.00	
no SMK & no DRK & no EXC	1.22	(1.08–1.38)
no SMK & DRK & EXC	1.05	(0.54–2.04)
SMK & no DRK & EXC	1.11	(0.89–1.39)
no SMK & DRK & no EXC	1.43	(0.90–2.28)
SMK & no DRK & no EXC	1.47	(1.19–1.81)
SMK & DRK & EXC	1.70	(1.23–2.36)
SMK & DRK & no EXC	1.68	(1.24–2.27)

* Adjusted by variables including age, sex, marital status, economic activity status, educational level, household income, medical aid, Charlson comorbidity index, the number of chronic diseases, body mass index, and medication adherence. Abbreviation: SMK smoking, DRK belonging to high-risk drinking group, EXC exercising moderate or over.

**Table 4:** Results of GEE analyzing the effect of practicing health behavior on unmet needs categorized by type of chronic diseases *.

Chronic Diseases	N (4 years)	Practicing Health Behavior		
		Yes	No	
			Unmet Needs	
			OR	95% CI
Type of chronic diseases ^a^	16,276			
Hypertension	3933	1.00	1.36	(1.04–1.79)
Hypertension & arthrosis	1987	1.00	1.11	(0.83–1.50)
Hypertension & dyslipidemia	1814	1.00	1.12	(0.75–1.66)
Arthrosis	1266	1.00	0.87	(0.62–1.23)
Hypertension & dyslipidemia & arthrosis	1118	1.00	0.98	(0.68–1.42)
Hypertension & diabetes mellitus	1062	1.00	2.40	(1.37–4.23)
Others ^b^	5096	1.00	1.42	(1.16–1.75)

* Adjusted by variables including age, sex, marital status, economic activity status, educational level, household income, medical aid, Charlson comorbidity index, the number of chronic diseases, body mass index, and medication adherence. ^a^: Type of chronic diseases was ordered by highest frequency among participants. ^b^: Less than 1000 participants were classified as belonging to the “Others” type of chronic disease.

## Data Availability

Publicly available datasets were analyzed in this study. This data can be found here: https://www.khp.re.kr:444/eng/main.do (accessed on 7 June 2021).
